# Surface Modification of Poly(lactic-co-glycolic acid) Microspheres with Enhanced Hydrophilicity and Dispersibility for Arterial Embolization

**DOI:** 10.3390/ma12121959

**Published:** 2019-06-18

**Authors:** Jiao Wang, Jianbo Li, Jie Ren

**Affiliations:** Institute of Nano and Biopolymeric Materials, Department of Polymeric Materials, Shanghai Key Laboratory for R&D and Application of Metalic Functional Materials, Key Laboratory of Advanced Civil Engineering Materials, Ministry of Education, School of Materials Science and Engineering, Tongji University, 4800 Caoan Road, Shanghai 201804, China; 1730615@tongji.edu.cn (J.W.); renjie6598@163.com (J.R.)

**Keywords:** functional, poly(lactic-co-glycolic acid), microspheres, surface modification

## Abstract

In this study, a series of poly(lactic-co-glycolic acid) (PLGA) microspheres with different particle sizes for arterial embolization surgery were prepared. The polydopamine (PDA) and polydopamine/polyethyleneimine (PDA/PEI) were respectively coated on the PLGA microspheres as shells, in order to improve the hydrophilicity and dispersibility of PLGA embolization microspheres. After modification, with the introduction of PDA and PEI, many hydrophilic hydroxyl and amine groups appeared on the surface of the PLGA@PDA and PLGA@PDA/PEI microspheres. SEM images showed the morphologies, sizes, and changes of the as-prepared microspheres. Meanwhile, the XPS and FT-IR spectra demonstrated the successful modification of the PDA and PEI. Water contact angles (WCAs) of the PLGA@PDA and PLGA@PDA/PEI microspheres became smaller, indicating a certain improvement in surface hydrophilicity. In addition, the results of in vitro cytotoxicity showed that modification had little effect on the biosafety of the microspheres. The modified PLGA microspheres suggest a promising prospective application in biomedical field, as the modified microspheres can reduce difficulties in embolization surgery.

## 1. Introduction

Functional polymeric microspheres, which are often used in chemical catalysis and adsorption, have some special advantages, including a large specific surface area, strong adsorption capacity, surface reaction ability, and ease of modification [1,2], tumor embolization [3,4], information transfer [5], chromatographic separation [6], and biomedicine [7]. Transcatheter arterial embolization (TAE) was first reported by Yamamoto et al. [8] in 1991, which was an effective technique for the insertion of a catheter into a target tissue or organ. The aim is to block the blood supply and nutrition of the target area. When the target tissue is a tumor, the tumor cells in the target area will be ischemic necrosis [9]. The embolic microspheres used in traditional TAE therapy were retained in the lesion for 8–12 months and could be washed out within 4 weeks. Embolization with embolic microspheres after the infusion of anticancer drugs can not only reduce drug clearance from cancer cells, but also induce ischemic necrosis [10]. The size of embolic microspheres is generally large and can be different depending on the site of embolization. Microspheres used in clinic include biodegradable and non-biodegradable materials [11,12]. Currently, biodegradable microspheres [13,14], such as polylactide (PLA) [15], have received wide attention.

As a naturally resourced biopolymer with good biocompatibility and biodegradability, PLA has attracted worldwide attention [16,17]. PLA has been widely researched and utilized in different fields, such as packaging materials [18], biomedicine [19] and automobiles [20]. The degradation rate of PLA-based microspheres can be controlled by adjusting the molecular structure and chemical composition, such as in the synthesis of poly(lactic-co-glycolic acid) (PLGA) [21]. However, PLA based materials have poor hydrophilicity [22]. After being prepared into microspheres, amphiphilic emulsifiers, such as polyvinyl alcohol (PVA), are washed away in post-process, leading to worse hydrophilicity and dispersion in the aqueous medium [23]. When operating vascular embolization in clinic, these poorly dispersed PLA-based microspheres cannot be dispersed evenly and stably in the injection or iodized oil developer. If they agglomerate, the hydrophobic microspheres block hypodermic needles or catheters, which could cause difficulties in operation.

To overcome this challenge, a mussel-inspired coating via a simple immersion method can be adopted [24,25]. Dopamine, a small molecule analog of the catechol, can carry out an oxidative self-polymerization under a mild alkaline environment (pH 8~8.5) and form uniform coatings on virtually any type or shape of surface, with great adhesive strength [26]. After the self-polymerization of dopamine, many hydrophilic hydroxyl groups are introduced to the surface of materials. In addition, the exposed and reactive hydroxyl groups of the polydopamine (PDA) coatings enable further functionalization on the surface through covalent grafting of polymers and the deposition of metal films via reduction from metal ions. This occurs because, when under slightly basic aqueous solutions, in the presence of oxygen, the catechol groups in dopamine can equilibrate to o-quinones that are extremely reactive to nucleophilic functional groups (e.g., amines and thiols) via a Schiff base or Michael addition reactions [27]. 

**Scheme 1:**
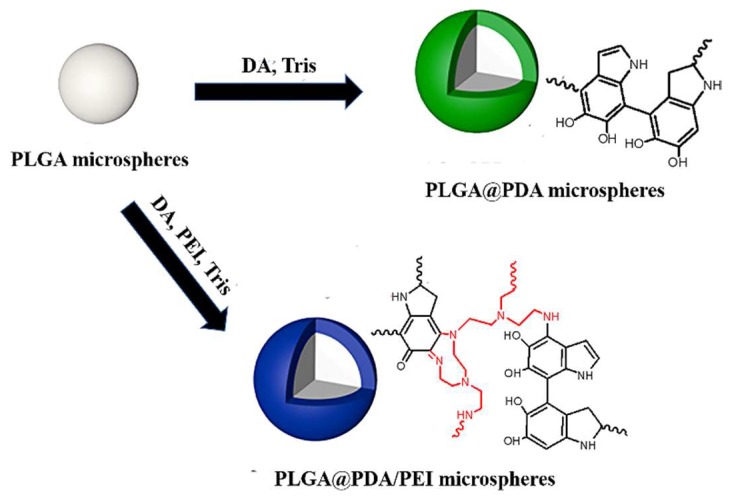
The fabrication process of poly(lactic-co-glycolic acid) (PLGA) @ polydopamine (PDA) and PLGA@PDA/polyethyleneimine (PEI) microspheres.

In this study, a series of PLGA microspheres with different particle sizes for arterial embolization surgery were prepared, and a simple coating method by using the versatile features of the PDA chemistry to increase the surface hydrophilicity and dispersion of the PLGA embolic microspheres is reported. This method utilized a simple immersion coating process to deposit PDA onto PLGA microspheres. Moreover, amino-rich polymer polyethyleneimine (PEI) [28,29] was used as a cross-linking component to promote the homogeneous polymerization of dopamine (DA) and the uniform co-deposition of PDA/PEI. The fabrication process of the functional microspheres is illustrated in Scheme 1, and the whole modification process was conducted in an aqueous solution, which avoided the pollution caused by the organic solvent. The method in this article was simple to implement and could be easily adapted for other substrates. 

## 2. Materials and Methods 

### 2.1. Materials

PLGA (molecular weight = 3.5 × 10^4^, 75/25) was purchased from Jinan Daigang Biomaterial Co., Ltd. (Jinan, Shandong, China). PVA was purchased from Aladdin Industrial Corporation (Shanghai, China). Dopamine hydrochloride, PEI (molecular weight = 600) and other reagents were provided by Sinopharm Chemical Reagent Co. Ltd. (Shanghai, China). All the chemicals were analytical grade and used without any further purification. The pH of Tris-buffer solution was adjusted to 8.5 using an already prepared 0.5 M NaOH solution.

### 2.2. Methods

#### 2.2.1. Synthesis of PLGA Microspheres

5 mg of PLGA was dissolved in 10 mL dichloromethane (DCM), and added into 20 mL PVA aqueous solution (concentration = 1%) dropwise. The system was kept sealed to avoid the DCM volatilizing. After stirring and emulsifying for 30 min, 6 mL PVA (concentration = 0.5%) was added. Then, the solution was left overnight at room temperature to evaporate the DCM completely. The solution was transferred into a 500 mL beaker and washed with deionized water for three times to remove the residual PVA. The crude microsphere was filtered out and dried through a freeze-drying method. Finally, zeolite was used to sieve the microspheres with different particle sizes. 

#### 2.2.2. Preparation of PLGA@PDA Core-Shell Microspheres

0.1 g of the as-prepared PLGA microspheres (300 µm) were dispersed in a 7 mL Tris-buffer solution (pH = 8.5, 50 mM) to wet them adequately for 15 min at room temperature. Then, 0.014 g of dopamine hydrochloride was added and stirred for 8 h. The products were separated and collected via centrifugation, washed with deionized water three times, and dried under a vacuum at 65 °C overnight.

#### 2.2.3. Preparation of PLGA@PDA/PEI Core-Shell Microspheres

Similar to the PLGA@PDA microspheres, to prepare PLGA@PDA/PEI microspheres, 0.1 g of PLGA microspheres (300 µm) were added in 7 mL Tris-buffer solution (pH = 8.5, 50 mM) to wet them adequately for 15 min at room temperature. Then, 0.014 g of dopamine hydrochloride and 0.014 g of PEI were added and stirred for 8 h. The products were centrifuged, washed with deionized water three times and dried under vacuum at 65 °C overnight.

#### 2.2.4. Materials Characterizations

The functional groups of the samples before and after modification were measured by an attenuated total reflection Fourier transform infrared (ATR-FTIR) spectroscopy (EQUIN0X55, BRUKER, Ettlingen, Germany) in the wavenumber range of 500–4000 cm^−1^. X-ray photoelectron spectroscopy (XPS) measurements were performed using a Thermo Scientific ESCALAB250 spectrometer (Thermo VG, Waltham, MA, USA) equipped with an Al-Kα X-ray source. The morphologies, sizes and morphology changes of the as-prepared microspheres samples were characterized by using the field-emission scanning electron microscope (FESEM, S-2360N, HITACHI Co., Tokyo, Japan) at 30 kV. 

#### 2.2.5. Evaluation of Hydrophilicity

Changes in water contact angle (WCA) were observed before and after surface modification by a contact angle tester (OCA20, Beijing Eastern-Dataphy Instruments Co. Ltd., Beijing, China). Before testing, double-sided sticky tape was stuck on the clean slides. Then, a few microspheres were compactly arranged on the surface of the tape and spread into about a 1 cm^2^ large region (approximately a drop of water dripping area). Placing the prepared slides on the contact angle tester, the test was conducted when drops of water just fell on the microspheres. The droplets were set to 12 µL and the test was carried out in air at ambient temperature.

#### 2.2.6. Cytotoxicity Assay in Vitro

To assess the cytotoxicity of the PLGA microspheres, PLGA@PDA microspheres, and PLGA@PDA/PEI microspheres, HepG2 cells were seeded in 36-well plates at a density of 8 × 10^3^ cells/well and treated with three kinds of microspheres under standard culture conditions using a complete medium for 24, 48, and 72 h, respectively. Cell viability was determined using the cell counting kit-8 (CCK-8) method in the HepG2 cell line. The group without microspheres treatment was used as a control, and cell viability for this group was considered to be 100%.

## 3. Results and Discussion

### 3.1. Morphological Characterization of PLGA Microspheres

**Figure 1:**
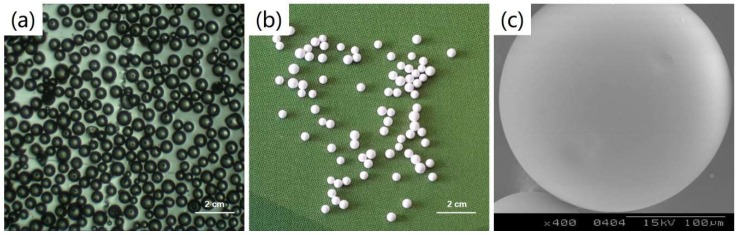
The optical micrograph (**a**), digital photograph (**b**), and SEM micrograph (**c**) of PLGA microspheres.

**Figure 2:**
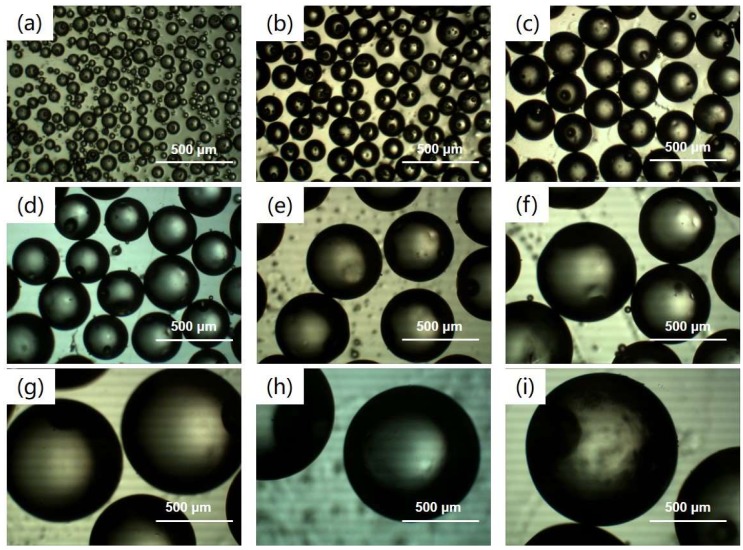
The optical micrographs of PLGA microspheres with different size fractions. (**a**) 0~200 µm; (**b**) 200~300 µm; (**c**) 300~400 µm; (**d**) 400~500 µm; (**e**) 500~600 µm; (**f**) 600~700 µm; (**g**) 700~800 µm; (**h**) 800~900 µm; (**i**) 900~1000 µm.

The optical micrograph (Figure 1a), digital photograph (Figure 1b), and SEM micrograph (Figure 1c) of the PLGA microspheres before surface modification are shown in Figure 1. The PLGA microspheres with different particle sizes were attained by using zeolite. The size distributions of the PLGA microspheres are shown in Figure 2. It is also evident from the images that the microspheres of the unfractionated sample had very different sizes, whereas the fractionated samples (Figure 2a–i) primarily contained microspheres within the specified ranges. According to the characteristics of individual patients and tumors, small embolic particle sizes (100–300 µm) [30] should be considered, because they can be injected into, or near, the tumors to achieve accurate drug delivery and embolization. Therefore, the as-prepared PLGA microspheres (300 µm) were modified.

### 3.2. Surface Morphology Analysis of Modified PLGA Microspheres

**Figure 3:**
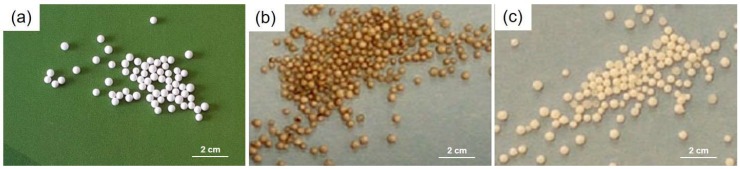
Color and size of (**a**) PLGA microspheres, (**b**) PLGA@PDA microspheres and (**c**) PLGA@PDA/PEI microspheres.

The color changes of the as-prepared microspheres before and after surface modification are clearly displayed in Figure 3. The PLGA microspheres were milky white (Figure 3a) originally. After surface coating, the PLGA@PDA microspheres became brown (Figure 3b), while the PLGA@PDA/PEI microspheres were light yellow (Figure 3c).

**Figure 4:**
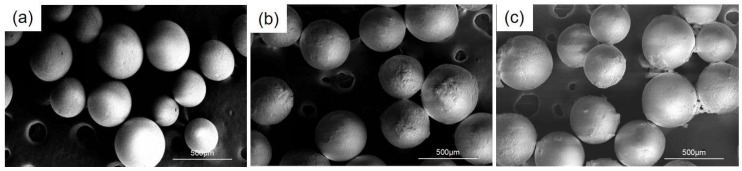
SEM micrographs of (**a**) PLGA microspheres, (**b**) PLGA@PDA microspheres, and (**c**) PLGA@PDA/PEI microspheres.

The surface morphologies and sizes of the bare PLGA, the PLGA@PDA, and the PLGA@PDA/PEI microspheres are displayed in Figure 4. From the SEM images, the PLGA microspheres (Figure 4a) showed smooth surfaces with diameters at 326 ± 25 µm. However, minor amounts of small particles were detected in the sieved fractions. This result is probably due to the hydrophobic nature of PLGA, which favors the adherence of small microparticles to larger ones during the sieving process. After modification, the surface of the PLGA@PDA and PLGA@PDA/PEI microspheres (Figure 4b,c) became rough, mainly because the self-aggregation of PDA can usually lead to particle-like morphology. In addition, the diameters of the PLGA@PDA and the PLGA@PDA/PEI microspheres were 386 ± 20 µm and 393 ± 25 µm, respectively, which indicated that the average thickness of the PDA and PDA/PEI layers were about 60~70 µm. Compared with the original PLGA microspheres, the structures of the modified microspheres displayed no obvious changes, indicating an appropriate method for surface modification with PDA.

### 3.3. Solubility in the Dichloromethane (DCM) of Modified PLGA Microspheres

**Figure 5:**
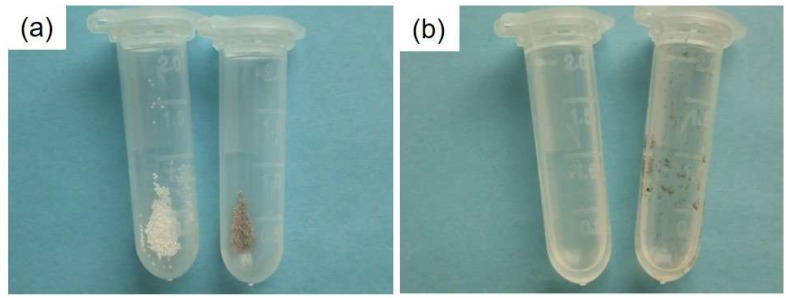
Corrosion of PLGA microspheres (**a**) and PLGA@PDA microspheres (**b**) in dichloromethane (DCM).

In generally, PLGA has a good solubility in dichloromethane (DCM) while the PDA’s solubility is poor. Figure 5 shows the experimental phenomena of PLGA and PLGA@PDA microspheres infiltrating into the DCM for the same amount of time. An equal amount of the PLGA, PLGA@PDA microspheres, and DCM were added into the centrifuge tubes to compare the corrosion of the two kinds of microspheres (Figure 5a). It could be clearly seen that the PLGA microspheres were dissolved completely, but the PLGA@PDA microspheres still dispersed in the DCM (Figure 5b). This phenomenon suggested that the self-polymerization of dopamine was successfully achieved on the surface of the PLGA microspheres.

### 3.4. FT-IR and XPS Spectra Analysis of Modified PLGA Microspheres

**Figure 6:**
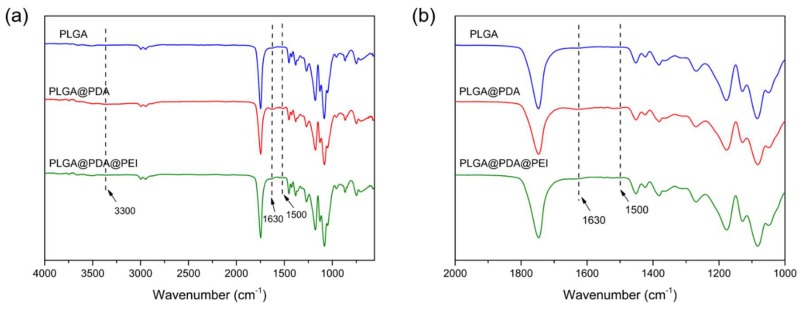
FTIR spectra with a scale of 4000~500 cm^−1^ (**a**) and 2000~1000 cm^−1^ (**b**) of the PLGA microspheres, PLGA@PDA microspheres, and PLGA@PDA/PEI microspheres.

FT-IR was employed to verify the surface composition of the surface coated microspheres (Figure 6). The signals that appeared at 3100~3000 cm^−1^ were assigned to the stretching vibrations of the C–H bond, and the peak intensities at 1600~1450 cm^−1^ and 880~680 cm^−1^ represented the bending vibrations of the C–H bond. The absorption peak at 1680–1630 cm^−1^, 1750~1735 cm^−1^, and 1210~1163 cm^−1^ were the stretching vibrations of the C=O bonds. Compared to the signals of PLGA, the signals of PLGA@PDA that appeared at 1500 cm^−1^ were assigned to the bending vibrations of the secondary amine bond, and the peak intensities at 1600 cm−1 represented the bending vibrations of the primary amine bond. This was because when dopamine is self-polymerized, a small amount of primary amine (-NH2) would appear, and part of the amine would become secondary ammonia (-NH). Further, the -NH peak also appeared at the same position of PLGA@PDA/PEI. Typical spectra at 1350~1000 cm^−1^ were due to the catechol hydroxyl group of the PDA coating, which indicated the formation of PDA coatings on the PLGA microspheres. PDA had hydroxyl groups, so there were wide small protrusions around 3300 cm^−1^ of the PLGA@PDA spectra. It was also demonstrated that a certain amount of PDA is coated on the PLGA. However, the hydroxyl region of the PLGA@PDA/PEI spectra was not obvious, because the addition of PEI made the hydroxyl groups on the original PDA crosslinked and the number of hydroxyl groups decreased. Therefore, the hydroxyl peak of the PLGA@PDA/PEI was lower than that of the PLGA@PDA. From the FT-IR spectra of the three as-prepared samples, there were some significant differences that proved the successful surface modification of the PLGA microspheres.

**Figure 7:**
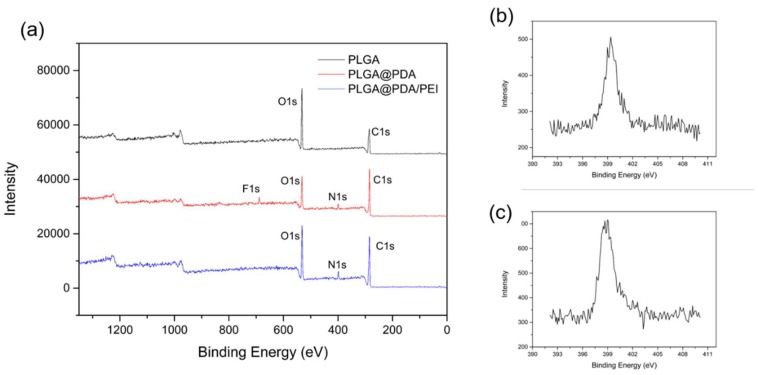
(**a**) The total XPS survey spectra of the PLGA microspheres, PLGA@PDA microspheres, and PLGA@PDA/PEI microspheres; (**b**) the N1s spectra of PLGA@PDA microspheres; (**c**) the N1s spectra of the PLGA@PDA/PEI microspheres.

XPS spectra were used to investigate the chemical compositions of the three as-prepared microspheres. The total XPS survey spectrum of the PLGA microspheres, the PLGA@PDA microspheres, and the PLGA@PDA/PEI microspheres is shown in Figure 7, which displays a significant difference of the three as-prepared samples, ranging from 0 to 1200 eV. The presence of the elements Carbon (C), Nitrogen (N), and Oxygen (O) in the PLGA@PDA microspheres and PLGA@PDA/PEI microspheres was evidenced by the photoelectron lines in the wide scan spectrum, with binding energies at 285, 400, and 531 eV, attributed to C1s, N1s, and O1s, respectively. Moreover, it could be observed that only elements of C and O were found in the sample of the bare PLGA microsphere. After surface modification, a new element, nitrogen (N), appeared (the N1s spectra corresponding to the NH- bonded to the carbon chain in the PDA), indicating the introduction of amino groups onto the surface of the PLGA microspheres. Moreover, as shown in Figure 7b,c, the intensity of N1s peaks experienced an obvious increase compared to the PLGA@PDA/PEI with PLGA@PDA microspheres, demonstrating the successful immobilization of the PDA and PEI. The element composition of C, N, and O in the PLGA microspheres, PLGA@PDA microspheres, and PLGA@PDA/PEI microspheres is shown in Table 1. Compared with the PLGA microspheres, the PLGA@PDA microspheres showed that the content of C and N increased while the content of O decreased, indicating that DA had been successfully grafted onto the surface of the PLGA microspheres. Similarly, the nitrogen content of the PLGA@PDA/PEI microspheres was higher than that of the PLGA@PDA microspheres, and the N/O and N/C increased from 0.08 and 0.232 to 0.107 and 0.287, respectively, indicating the success of the PEI grafting.

### 3.5. The Water Contact Angles of Modified PLGA Microspheres

**Figure 8:**
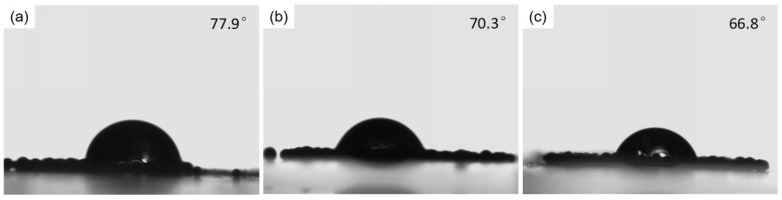
The water contact angles (WCAs) of (**a**) PLGA microspheres, (**b**) PLGA@PDA microspheres, and (**c**) PLGA@PDA/PEI microspheres.

The aim of surface modification is to introduce hydrophilic amino groups and increase the surface energy of the PLGA microspheres. Figure 8 shows the WCA in the air of the PLGA, PLGA@PDA, and PLGA@PDA/PEI microspheres. Under the same conditions, the WCA changed from 77.9° to 70.3° after being homogeneously coated with polydopamine. Meanwhile, the WCA of the PLGA@PDA/PEI microspheres had a remarkable decrease from 77.9° to 66.8°, displaying a better hydrophilicity. The changes in the surface wettability of the as-prepared microspheres indicated that it was successfully coated with PDA and grafted with PEI on the surface.

### 3.6. In-Vitro Cytotoxicity Assessment of Modified PLGA Microspheres

**Figure 9:**
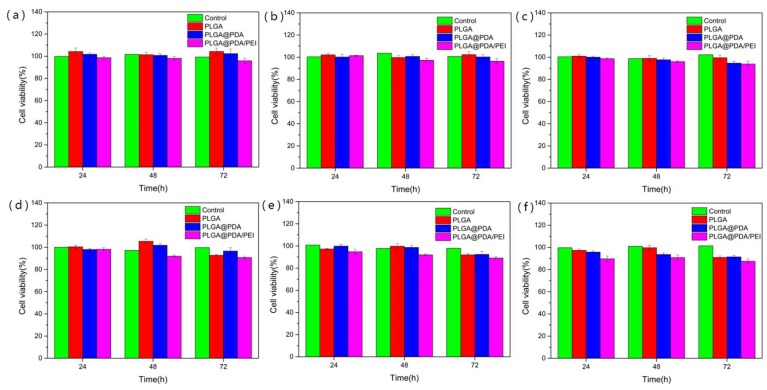
Cell viability after treatment with blank PLGA, PLGA@PDA and PLGA@PDA/PEI microspheres at the concentration of (**a**) 37.25 μg/mL; (**b**) 62.5 μg/mL; (**c**) 125 μg/mL; (**d**) 250 μg/mL; (**e**) 500 μg/mL and (**f**) 1000 μg/mL.

HepG2 cells were used to test the in-vitro cytotoxicity of three kinds of microspheres, and relevant results are illustrated in Figure 9. The viability of cells was higher than 90% after 24 h incubation in the presence of three microspheres. When the cells were incubated for 48 h, a similar situation was observed. The viability of cells decreased slightly but still reached around 90% or higher when the incubation time was extended up to 72 h, indicating that the modification had little effect on the cytotoxicity of the PLGA microspheres. The viability of all cells was around 90% or higher in the presence of different kinds of concentrations of modified PLGA microspheres. The cell viability after treatment with PLGA@PDA/PEI microspheres at a concentration of 1000 μg/mL for 72 h was the lowest, but it was still about 89%. This result showed that the concentration of three kinds of microspheres had little effect on cytotoxicity. In view of the high viability of HepG2 cells, the results of the cytotoxicity test verify that PLGA microspheres, PLGA@PDA microspheres, and PLGA@PDA/PEI microspheres are nearly nontoxic to normal cells.

## 4. Conclusions

This study reported an eco-friendly preparation of biodegradable and biocompatible PLGA@PDA and PLGA@PDA/PEI microspheres with core-shell structures via a simple immersion method. The PLGA microspheres formed by mechanical stirring were coated with PDA and PDA/PEI shell layers of 60~70 µm thickness. Comprehensive characterizations, including SEM images, FT-IR, and XPS spectra of the PLGA, PLGA@PDA, and PLGA@PDA/PEI microspheres confirmed successful surface coating and showed changes in the colors and sizes of the three as-prepared microspheres, as well. The introduction of hydrophilic groups made the WCA of the microspheres become small, leading to the monodispersity of the two modified microspheres in the aqueous solution. Moreover, the PLGA microspheres remained nontoxic before and after surface modification. The combination of excellent biocompatibility, favorable hydrophilicity, and dispersion in an aqueous medium makes these microspheres a promising prospect for application in embolization surgery. Researches focused on embolic microspheres will enable the development of new TAE materials for different diseases and discover more applications in biomedicine.

## Figures and Tables

**Table 1:** The chemical element composition of C, N, and O in the PLGA Microspheres, PLGA@PDA microspheres, and PLGA@PDA/PEI microspheres.

Samples	Content/%			Relative Content		
	C	O	N	O/C	N/C	N/O
PLGA	61.2	38.8	-	0.634	0	0
PLGA@PDA	68.42	23.57	5.46	0.344	0.080	0.232
PLGA@PDA/PEI	67.6	25.18	7.22	0.372	0.107	0.287

## References

[1] Du W.L., Niu S.S., Xu Z.R., Xu Y.L. (2009). Preparation, Characterization, and Adsorption Properties of Chitosan Microspheres Crosslinked by Formaldehyde for Copper (II) from Aqueous Solution. J. Appl. Polym. Sci..

[2] Yoshida S., Kikuchi S., Kanehashi S., Okamoto K., Ogino K. (2018). Microfluidic Fabrication of Morphology-Controlled Polymeric Microspheres of Blends of Poly(4-butyltriphenylamine) and Poly(methyl methacrylate). Materials.

[3] Fuchs K., Duran R., Denys A., Bize P.E., Borchard G., Jordan O. (2017). Drug-eluting embolic microspheres for local drug delivery-State of the art. J. Control. Release.

[4] Porcu E.P., Salis A., Rassu G., Maestri M., Galafassi J., Bruni G., Gavini E. (2017). Engineered polymeric microspheres obtained by multi-step method as potential systems for transarterial embolization and intraoperative imaging of HCC: Preliminary evaluation. Eur. J. Pharm. Biopharm..

[5] Zhao Q., Piao J.F., Peng W.P., Wang Y., Zhang B., Gong X.Q., Chang J. (2018). Simple and Sensitive Quantification of MicroRNAs via PS@Au Microspheres-Based DNA Probes and DSN-Assisted Signal Amplification Platform. ACS Appl. Mater. Interfaces.

[6] Lai S.Z., Ouyang X.L., Cai C.Q., Xu W.S., Chen C.Y., Chen X.M. (2017). Surface-imprinted microspheres prepared by a template-oriented method for the chiral separation of amlodipine. J. Sep. Sci..

[7] Saralidze K., Koole L.H., Knetsch M.L.W. (2010). Polymeric Microspheres for Medical Applications. Materials.

[8] Yamamoto T., Kawarada Y., Yano T. (1991). Spontaneous rupture of hemangioma of the liver: Treatment with transcatheter hepatic arterial embolization. Am. J. Gastroenterol..

[9] Lencioni R., de Baere T., Soulen M.C., Rilling W.S., Geschwind J.F.H. (2016). Lipiodol Transarterial Chemoembolization for Hepatocellular Carcinoma: A Systematic Review of Efficacy and Safety Data. Hepatology.

[10] Ozyer U. (2017). Transcatheter Arterial Embolization with N-Butyl-2-Cyanoacrylate in the Management of Spontaneous Hematomas. Cardiovasc. Interv. Radiol..

[11] Poursaid A., Jensen M.M., Huo E., Ghandehari H. (2016). Polymeric materials for embolic and chemoembolic applications. J. Control. Release.

[12] Lin G.Q., Chen H.Y., Zhou H.J., Zhou X.H., Xu H. (2018). Preparation of Tea Tree Oil/Poly(styrene-butyl methacrylate) Microspheres with Sustained Release and Anti-Bacterial Properties. Materials.

[13] Xie Y., Ma X.X., Liu X.J., Long Q.M., Wang Y., Yao Y.W., Cai Q. (2018). Carrier-Free Microspheres of an Anti-Cancer Drug Synthesized via a Sodium Catalyst for Controlled-Release Drug Delivery. Materials.

[14] Bee S.L., Hamid Z.A.A., Mariatti M., Yahaya B.H., Lim K., Bee S.T., Sin L.T. (2018). Approaches to Improve Therapeutic Efficacy of Biodegradable PLA/PLGA Microspheres: A Review. Polym. Rev..

[15] Zhu Z., Min T.T., Zhang X.J., Wen Y.Q. (2019). Microencapsulation of Thymol in Poly(lactide-*co*-glycolide) (PLGA): Physical and Antibacterial Properties. Materials.

[16] Garlotta D. (2001). A literature review of poly(lactic acid). J. Polym. Environ..

[17] Zheng X., Li H., He Y., Yuan M., Shen M., Yang R., Yang C. (2019). Preparation and In Vitro Release of Total Alkaloids from Alstonia Scholaris Leaves Loaded mPEG-PLA Microspheres. Materials.

[18] Arrieta M.P., Samper M.D., Aldas M., Lopez J. (2017). On the Use of PLA-PHB Blends for Sustainable Food Packaging Applications. Materials.

[19] Pellis A., Silvestrini L., Scaini D., Coburn J.M., Gardossi L., Kaplan D.L., Guebitz G.M. (2017). Enzyme-catalyzed functionalization of poly(l-lactic acid) for drug delivery applications. Process Biochem..

[20] Notta-Cuvier D., Odent J., Delille R., Murariu M., Lauro F., Raquez J.M., Dubois P. (2014). Tailoring polylactide (PLA) properties for automotive applications: Effect of addition of designed additives on main mechanical properties. Polym. Test..

[21] Xu Y.H., Kim C.S., Saylor D.M., Koo D. (2017). Polymer degradation and drug delivery in PLGA-based drug-polymer applications: A review of experiments and theories. J. Biomed. Mater. Res. Part B Appl. Biomater..

[22] Wu D., Samanta A., Srivastava R.K., Hakkarainen M. (2018). Nano-Graphene Oxide Functionalized Bioactive Poly(lactic acid) and Poly(epsilon-caprolactone) Nanofibrous Scaffolds. Materials.

[23] Sabee M., Kamalaldin N.A., Yahaya B.H., Hamid Z.A.A. (2016). Characterization and In Vitro Study of Surface Modified PLA Microspheres Treated with NaOH. J. Polym. Mater..

[24] Liao M.H., Wan P.B., Wen J.R., Gong M., Wu X.X., Wang Y.G., Zhang L.Q. (2017). Wearable, Healable, and Adhesive Epidermal Sensors Assembled from Mussel-Inspired Conductive Hybrid Hydrogel Framework. Adv. Funct. Mater..

[25] Kao C.T., Chen Y.J., Ng H.Y., Lee A.K.X., Huang T.H., Lin T.F., Hsu T.T. (2018). Surface Modification of Calcium Silicate via Mussel-Inspired Polydopamine and Effective Adsorption of Extracellular Matrix to Promote Osteogenesis Differentiation for Bone Tissue Engineering. Materials.

[26] Ryu J.H., Messersmith P.B., Lee H. (2018). Polydopamine Surface Chemistry: A Decade of Discovery. ACS Appl. Mater. Interfaces.

[27] Zhan Y.Q., Wan X.Y., He S.J., Yang Q.B., He Y. (2018). Design of durable and efficient poly(arylene ether nitrile)/bioinspired polydopamine coated graphene oxide nanofibrous composite membrane for anionic dyes separation. Chem. Eng. J..

[28] Zhao J., Fang C.H., Zhu Y.W., He G.W., Pan F.S., Jiang Z.Y., Wang B.Y. (2015). Manipulating the interfacial interactions of composite membranes via a mussel-inspired approach for enhanced separation selectivity. J. Mater. Chem. A.

[29] Zhan Z.W., Zhang X.X., Huang J.Y., Huang Y., Huang Z.W., Pan X., Wu C.B. (2017). Improved Gene Transfer with Functionalized Hollow Mesoporous Silica Nanoparticles of Reduced Cytotoxicity. Materials.

[30] Fateen W., Khan F., O’neill R.J. (2017). Healthcare costs of transarterial chemoembolization in the treatment of hepatocellular carcinoma. J. Hepatocell. Carcinoma.

